# Myogenic exosome miR-140-5p modulates skeletal muscle regeneration and injury repair by regulating muscle satellite cells

**DOI:** 10.18632/aging.205617

**Published:** 2024-02-29

**Authors:** Xiaorui Cao, Linli Xue, Xiuju Yu, Yi Yan, Jiayin Lu, Xiaomao Luo, Haidong Wang, Juan Wang

**Affiliations:** 1 College of Veterinary Medicine, Shanxi Agricultural University, Taigu, Shanxi 030801, China; 2 Department of Nephrology, Shanghai General Hospital, Shanghai Jiaotong University School of Medicine, Shanghai 200080, China

**Keywords:** exosomes, miR-140-5p, muscle satellite cells, muscle injury repair, regeneration

## Abstract

Muscle satellite cells (SCs) play a crucial role in the regeneration and repair of skeletal muscle injuries. Previous studies have shown that myogenic exosomes can enhance satellite cell proliferation, while the expression of miR-140-5p is significantly reduced during the repair process of mouse skeletal muscle injuries induced by BaCl2. This study aims to investigate the potential of myogenic exosomes carrying miR-140-5p inhibitors to activate SCs and influence the regeneration of injured muscles. Myogenic progenitor cell exosomes (MPC-Exo) and contained miR-140-5p mimics/inhibitors myogenic exosomes (MPC-Exo^140+^ and MPC-Exo^140−^) were employed to treat SCs and use the model. The results demonstrate that miR-140-5p regulates SC proliferation by targeting *Pax7*. Upon the addition of MPC-Exo and MPC-Exo^140−^, *Pax7* expression in SCs significantly increased, leading to the transition of the cell cycle from G1 to S phase and an enhancement in cell proliferation. Furthermore, the therapeutic effect of MPC-Exo^140−^ was validated in animal model, where the expression of muscle growth-related genes substantially increased in the gastrocnemius muscle. Our research demonstrates that MPC-Exo^140−^ can effectively activate dormant muscle satellite cells, initiating their proliferation and differentiation processes, ultimately leading to the formation of new skeletal muscle cells and promoting skeletal muscle repair and remodeling.

## INTRODUCTION

Skeletal muscle, an essential component of the human body, plays a crucial role in various functions, such as movement, metabolism, and endocrine regulation. Its regenerative capacity and plasticity rely on muscle satellite cells found within the skeletal muscle [1–3]. In the event of muscle injury, these quiescent satellite cells become activated, entering the cell cycle, proliferating, and differentiating to repair the damaged muscle and replenish the cell pool [4–6]. One of the key markers of muscle satellite cells is the expression of the paired box (*Pax7*), which is vital for the survival, proliferation, and prevention of premature differentiation of myogenic progenitor cells. *Pax7* acts as an upstream gene of myoblast determination protein 1 (*MyoD*) and *MRF5*, initiating the expression of muscle-related genes and initiating the myogenic program. The absence of *Pax7* and its homolog *PAX3* leads to the death or differentiation of satellite cells into nonmuscle cell types, such as cartilage [7–10].

Studies have demonstrated that specific microRNAs (miRNAs), such as miR-1, miR-206, miR-133, and miR-664-5p, can regulate the proliferation and differentiation of muscle satellite cells by modulating the expression of *Pax7* and serum response factor [11, 12], after inhibiting the expression of miR-140-5p in blood vessels and cardiac muscles, it significantly inhibits atherosclerosis and acute myocardial infarction [13]. These findings underscore the critical role of miRNAs in regulating myocyte functions, including proliferation, differentiation, contraction, and stress response, through the regulation of gene expression [14]. While most of these studies have been conducted at the cellular level, the exogenous addition of miRNA mimics or inhibitors to living animals is challenging due to rapid degradation by ribonucleases in the plasma. Additionally, viral-mediated delivery carries the risk of integrating gene fragments into host cell chromosomes, potentially leading to cancer development [15]. Therefore, the development of a delivery system that can protect miRNAs from degradation and facilitate their cellular uptake, intracellular release, and downstream gene action is essential for promoting muscle development and injury repair.

Extracellular vesicles, including exosomes, microvesicles, and apoptotic bodies, are double-membrane structures secreted by cells and serve as novel delivery carriers [16]. Among these, exosomes, with a diameter of 50–150 nm, play a crucial role in intercellular communication throughout the body. They can carry and deliver a diverse range of cargoes, including metabolites, short-chain nucleic acids, amino acids, mRNA, and proteins [17, 18]. Emerging research suggests that muscle, as an endocrine organ, secretes exosomes that contribute to muscle growth, development, regeneration after injury or chronic disease, as well as muscle atrophy and aging [19–21]. Furthermore, exosomes facilitate communication between skeletal muscle and other tissues [3]. By selectively packaging miRNAs, exosomes can deliver them to recipient cells, modulating the expression of target genes and influencing the functionality of the recipient cells [22–24].

Based on these findings, it is hypothesized that myogenic exosomes carrying specific miRNAs, when injected into muscles, can regulate muscle satellite cells within the muscle, thereby affecting muscle injury repair. In this study, we aimed to produce and characterize engineered exosomes containing miR-140-5p mimics or inhibitors. Through *in vitro* experiments and acute injury models, we will coculture or inject these engineered exosomes, monitoring the activation and proliferation of muscle satellite cells. By evaluating the impact of engineered myogenic exosomes on muscle injury repair, we aim to provide a novel therapeutic strategy for the treatment of muscle injuries.

## RESULTS

### miR-140-5p is significantly reduced in the gastrocnemius muscle following acute injury, contrasting with the expression trend of Pax7

**Figure 1:**
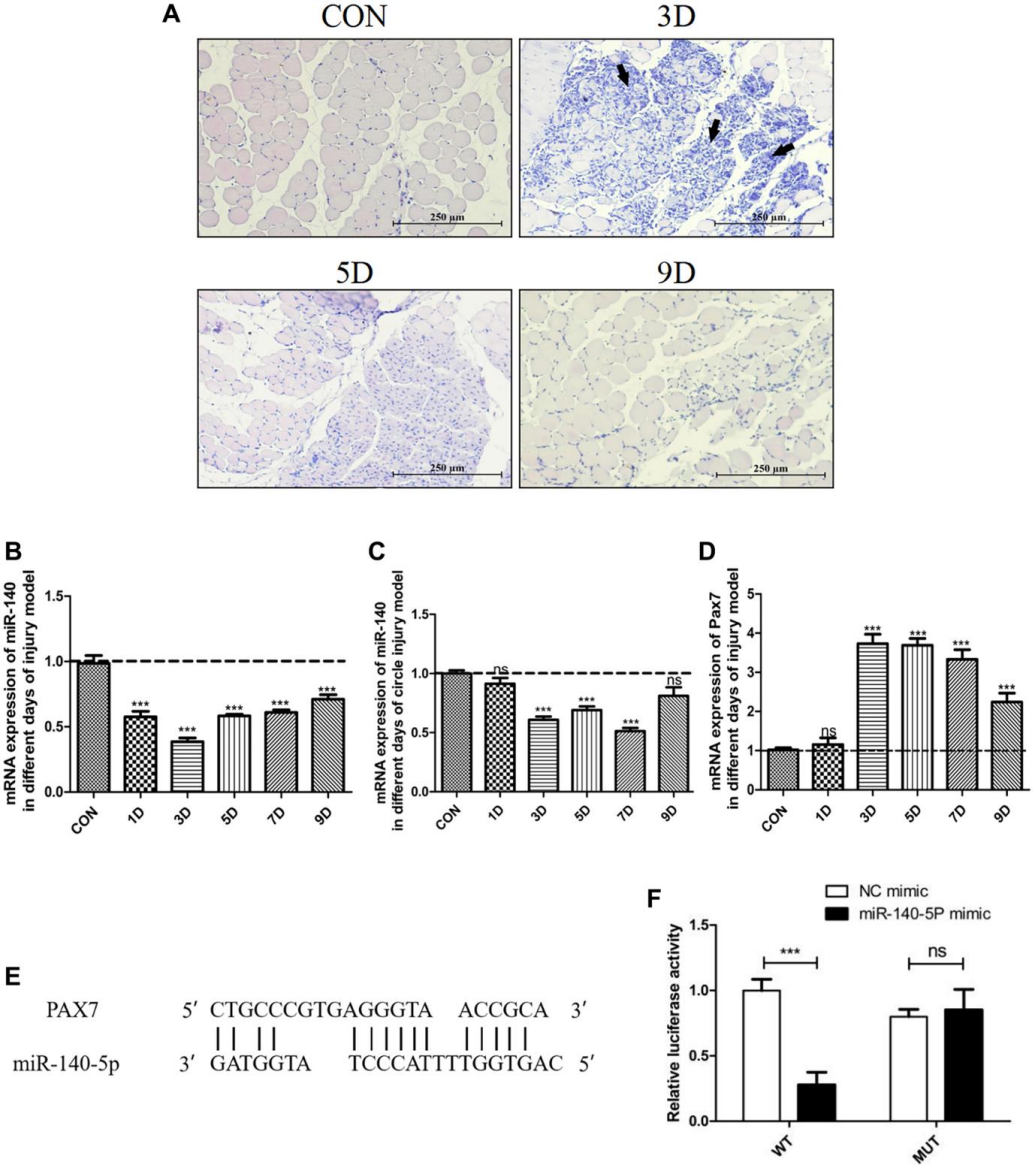
**Significant downregulation of miR-140-5p expression following injury, contrasting with the expression trend of *Pax7*.** Male wildtype C57BL/6 mice established damage model by intramuscular injection of 1.2% BaCl_2_ into the gastrocnemius muscle. (**A**) displays the muscle repair and regeneration process post-acute damage to the gastrocnemius muscle caused by BaCl_2_, as shown through histological examination staining (Scale bar in 250 μm). (**B**–**D**) present the RT-PCR results (fold change) illustrating the expression trends of miR-140-5p and *Pax7* in the gastrocnemius muscle and circulation at different time points postinjury (*n* = 4 biological replicates). (**E**, **F**) demonstrate the binding pattern between miR-140-5p and *Pax7*, along with the dual luciferase assay results (*n* = 4), where WT refers to the wild type and MUT to the mutant type. The results are reported in the line/bar graph as mean ± SE; *n* = 4/group; different letters between bars mean *P* ≤ 0.05 analyses followed by non-paired Student’s *t*-test. ^*^P < 0.05; ^**^P < 0.01; ^***^P < 0.001 vs. CON Group.

In a previous chronic kidney disease model, ligation of a single ureter not only resulted in renal fibrosis but also led to skeletal muscle atrophy, accompanied by a decrease in the expression of miR-140-5p in plasma exosomes [24]. To investigate the changes in miR-140-5p during muscle injury, an acute damage model was established by intramuscular injection of BaCl_2_ into gastrocnemius muscle. Gastrocnemius muscle and blood samples were collected at various time points (days 1, 3, 5, 7, and 9) postinjection. Histological examination (Figure 1A) revealed that compared to normal mice, the injury group exhibited severe muscle fiber damage on day 3, with visible cell fragments in the interstitial spaces and prominent infiltration of inflammatory cells. By day 5, central nuclei were observed in many muscle fibers indicating that the muscles were in the process of repair, and by day 9, these central nuclei had shifted toward the cell periphery, indicating the final stage of repair.

The expression of miR-140-5p was measured in the gastrocnemius muscle and circulating blood of the injury model at different time points. Compared to control mice, miR-140-5p expression significantly decreased in both the gastrocnemius muscle and circulating blood on days 1–5 following BaCl_2_ injury, gradually increasing on days 7–9. In contrast, the expression trend of the muscle satellite cell marker gene *Pax7* was opposite to that of miR-140-5p (Figure 1B–1D).

To explore the role of miR-140-5p in skeletal muscle injury, potential downstream target genes were predicted, and *Pax7* was identified as a target gene of miR-140-5p (Figure 1E). A dual-luciferase reporter assay confirmed that the luciferase activity of the *Pax7* wild-type 3′UTR was significantly inhibited by miR-140-5p mimics compared to the negative control group (*P* < 0.05). However, there was no significant difference in luciferase activity when using the mutant 3′UTR (*P* > 0.05). This indicates that miR-140-5p specifically binds to the *Pax7*-3′UTR and downregulates *Pax7* gene expression at the posttranscriptional level (Figure 1F).

### Preparation and characterization of engineered MPC-EVs

To investigate the role of miR-140-5p in the regeneration process following skeletal muscle injury, this study employed myogenic progenitor cell exosomes (MPC-Exo) as a delivery system for miR-140-5p. Engineered extracellular vesicles carrying miR-140-5p mimics and inhibitors were constructed to selectively deliver the miRNAs to target muscle cells.

**Figure 2:**
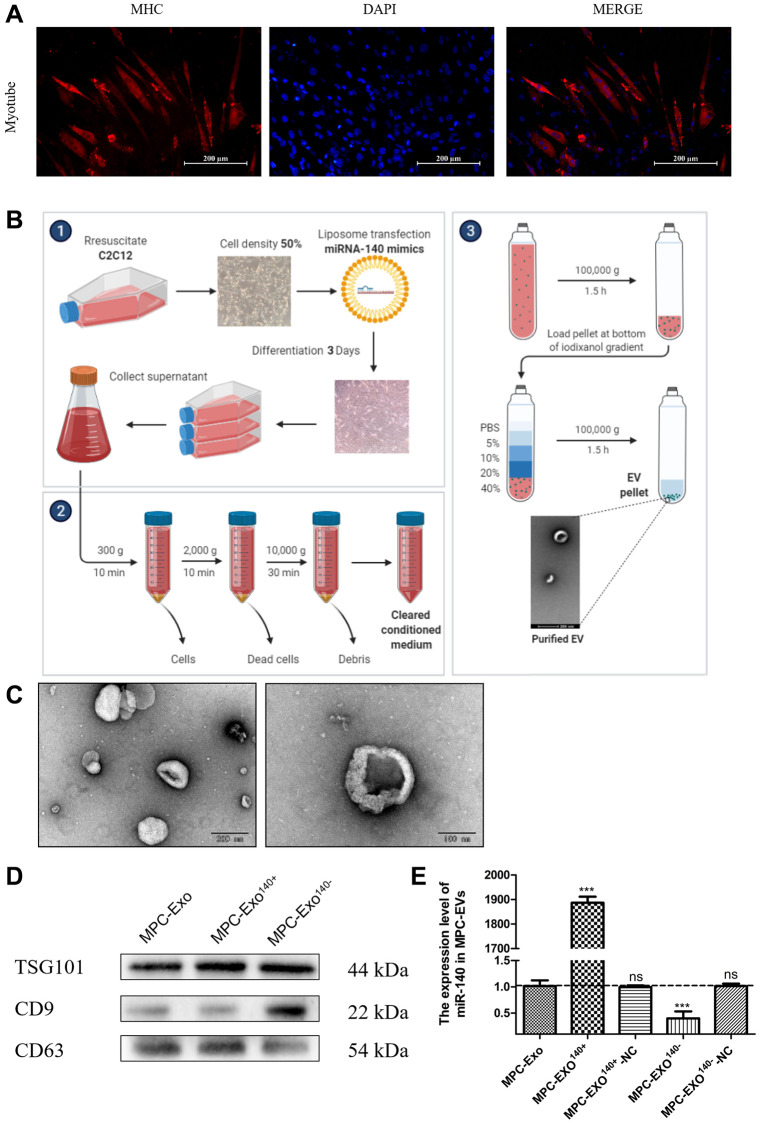
**Production and identification engineered exosomes containing miR-140-5p mimics or inhibitors.** Preparation and identification of MPC-EVs. (**A**) illustrates the formation of myotubes post differentiation of C2C12 cells, shown via IF with red representing the heavy chain myosin (MYH) and blue for DAPI-stained nuclei (Scale bar in 200 μm). (**B**) depicts the preparation process of MPC-EVs. (**C**) presents the TEM results, showing the characteristic cup-shaped or semispherical morphology of the myogenic extracellular vesicles, in agreement with the expected bilayer membrane ultrastructure of extracellular vesicles. (**D**) shows WB results indicating positive reactions for the marker proteins TSG101, CD9, and CD63 in all types of MPC-EVs. (**E**) illustrates RT-PCR results (fold change), showing the miR-140-5p levels in MPC-Exo^140+^ and MPC-Exo^140−^, which are 1800 times and 1/3 of normal extracellular vesicles, respectively. MPC-Exo^140+^-NC and MPC-Exo^140−^-NC showed no difference from normal extracellular vesicles. Different letters between bars mean *P* ≤ 0.05 analyses followed by non-paired Student’s *t*-test. ^ns^*p* > 0.05, ^*^p < 0.05, ^**^p < 0.01 and ^***^p < 0.001 vs. MPC-Exo.

Mouse myoblasts were transfected with miR-140-5p mimics and inhibitors using liposomes and subsequently underwent differentiation to form myotubes (Figure 2A). After a 36-hour starvation period, the supernatant was collected, and MPC-Exos were isolated through gradient centrifugation (Figure 2B). The isolated MPC-Exo, MPC-Exo^140+^, and MPC-Exo^140−^ were characterized using transmission electron microscopy (TEM), nanoparticle tracking analysis, and Western blot (WB). TEM observations confirmed the uniform physical appearance of the MPC-Exos (Figure 2C). Nanoparticle tracking analysis revealed peak diameter sizes of 130.1 nm, 130.9 nm, and 128.9 nm for MPC-Exo, MPC-Exo^140+^, and MPC-Exo^140−^, respectively, with particle abundances of 2.3E+10 particles/mL, 2.2E+10 particles/mL, and 2.3E+10 particles/mL. These findings indicated that the transfection process did not affect the yield of extracellular vesicles. Furthermore, Western blot analysis confirmed the presence of the exosome marker proteins TSG101, CD9, and CD63 (Figure 2D).

The experimental results demonstrated that the content of miR-140-5p in MPC-Exo^140+^ and MPC-Exo^140−^ was 1800-fold and one-third of that in normal exosomes, respectively. The content of miR-140-5p in MPC-EXO-Mimics-NC and MPC-EXO-Inhibitor-NC did not differ significantly from that in normal exosomes, indicating the successful preparation of engineered exosomes overexpressing and suppressing miR-140-5p, respectively (Figure 2E).

### Activation of satellite cells by MPC-Exo and the interference of Pax7 expression by MPC-Exo^140−^, promoting satellite cell proliferation

**Figure 3:**
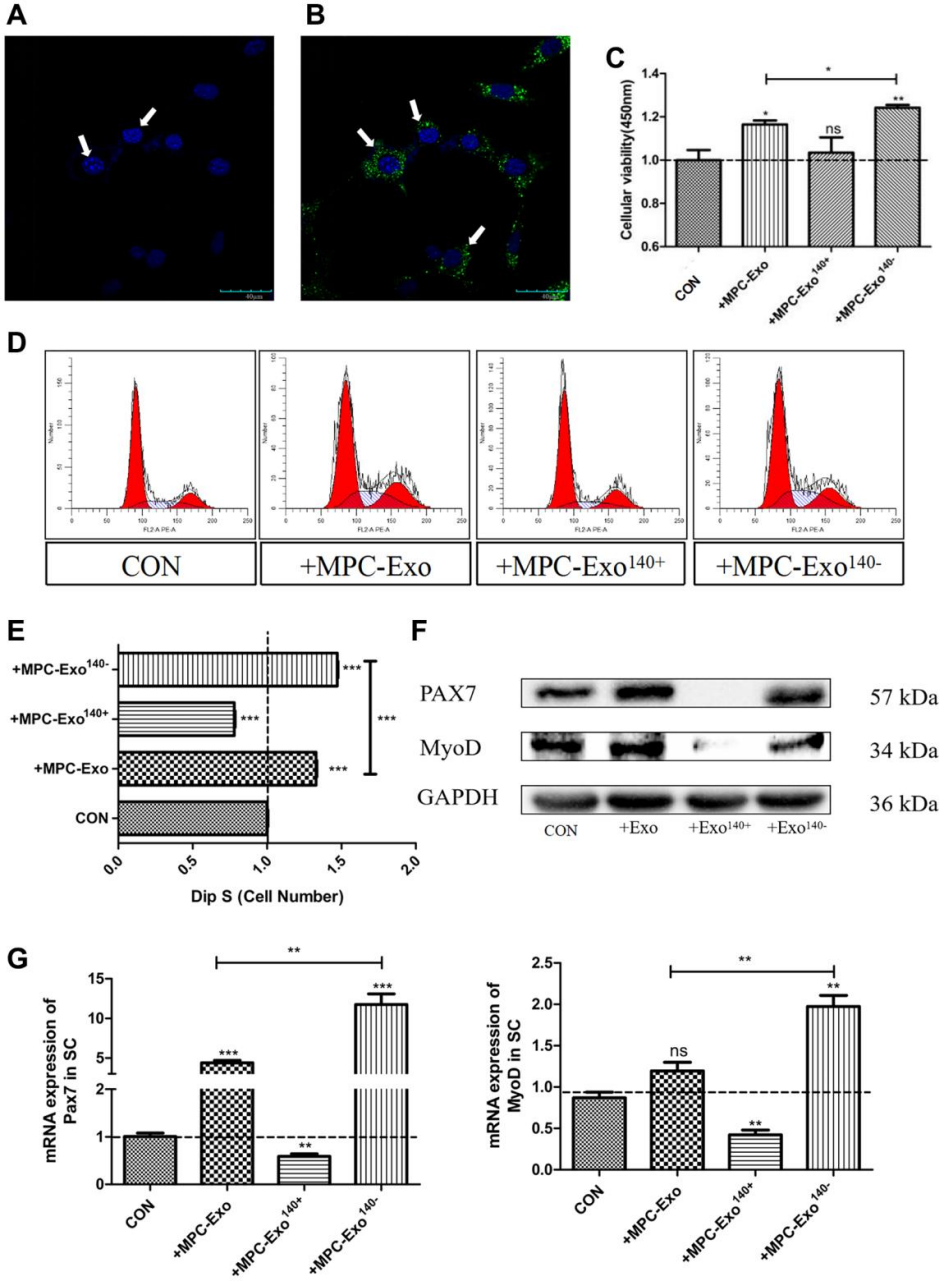
**MPC-EVs and MPC-Exo^140−^ internalized by SCs induced *Pax7* and *MyoD* significantly increased and promoted satellite cell proliferation.** Activation of muscle satellite cells by MPC-EVs and MPC-Exo^140−^. The process of SCs taking up myogenic extracellular vesicles was displayed under a live-cell confocal microscopy workstation, with (**A**) SC without MPC-Exond (**B**) treated with MPC-Exo^140-^ green fluorescence indicates uptake myogenic extracellular vesicles (Scale bar in 40 μm). (**C**) shows CCK8 results indicating a significant increase in cell proliferation after coincubation of SCs with MPC-EVs, especially in the MPC-Exo^140−^ group. (**D**, **E**) present the results of cell cycle analysis by flow cytometry, revealing a significant increase in the number of cells in the S phase in the MPC-Exo^140−^ group, while a significant decrease is seen in the MPC-Exo^140+^ group. (**F**, **G**) show WB and RT-PCR results (fold change), showing that the expression of *Pax7* and *MyoD* significantly increased in the MPC-Exo^140−^ group, while they were significantly inhibited in the MPC-Exo^140+^ group. Data: by one-way ANOVA analysis. ^ns^*p* > 0.05, ^*^p < 0.05, ^**^p < 0.01 and ^***^p < 0.001 vs. SC.

To investigate the effects of engineered muscle-derived extracellular vesicles *in vitro*, satellite cells were isolated and purified from mouse skeletal muscle [23] and then cocultured with MPC-Exo, MPC-Exo^140+^, and MPC-Exo^140−^. First, the muscle-derived extracellular vesicles were labeled with PKH67, and the uptake process of the vesicles by satellite cells was observed at different time points using live-cell imaging. The results showed a gradual increase in the number of PKH67-labeled muscle-derived extracellular vesicles within the satellite cells over time, indicating the internalization of MPC-Exos by satellite cells (Figure 3A, 3B).

Next, the viability of satellite cells after cocultivation with different engineered extracellular vesicles was assessed using a CCK8 cell proliferation assay. The results demonstrated significant differences in satellite cell proliferation after cocultivation with MPC-Exo compared to normal satellite cells (*p* < 0.05) and notably significant differences after cocultivation with MPC-Exo^140−^ (*p* < 0.01) (Figure 3C). Flow cytometry analysis of the cell cycle revealed that cocultivation with MPC-Exo^140−^ significantly increased the percentage of satellite cells in the S phase (25.05%) compared to normal satellite cells (17.02%) and showed a significant increase compared to cocultivation with MPC-Exo (22.62%) (Figure 3D, 3E). Furthermore, WB and qPCR assays were performed, indicating that the expression levels of *Pax7* and *MyoD* significantly increased after cocultivation, suggesting the activation of satellite cells by MPC-Exo and the interference of *Pax7* expression by MPC-Exo^140−^, promoting satellite cell proliferation (Figure 3F, 3G).

**Figure 4:**
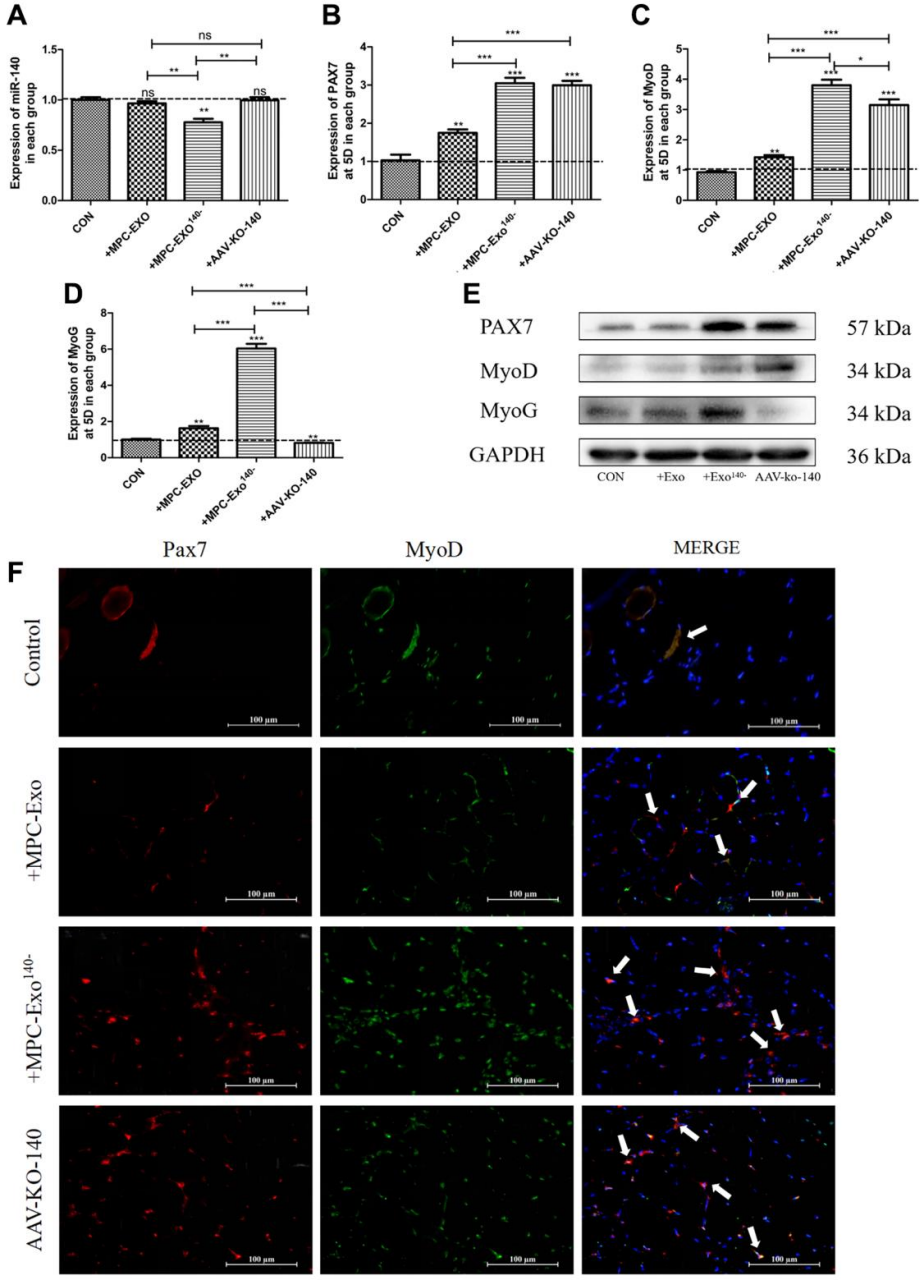
**Muscle satellite cells in the gastrocnemius muscle activate by MPC-Exo, MPC-Exo^140-^ or AAV-KO-140.** Eight-week-old male wildtype C57BL/6 mice were treated with MPC-Exo and MPC-Exo^140−^ 3 times. Five-week-old mice were injected with adeno-associated virus carrying miR-140-5p inhibitors. RT-qPCR was used to detect the expression (fold change) of miR-140-5p (**A**), *Pax7* (**B**), MyoD (**C**) and MyoG (**D**). The amount of proteins for Pax 7, MyoD and myoD was analyzed by Western blot (**E**). Data: mean ± SEM (*N* = 5). ^*^P < 0.05 by one-way ANOVA analysis. (**F**) presents the IF results: activated SCs are characterized by the coexpression of *Pax7^+^*/*MyoD^+^*. The marked locations in the figure indicate that in the control group, these cells are expressed only in the cell pool to maintain stem cell numbers. In the injected MPC-Exo group, there was a significant increase in activated SCs around the muscle fibers, indicating that muscle satellite cells were in a proliferative state. This proliferation of activated SCs was further augmented in the injected MPC-Exo^140−^ group and AAV-KO-140 group (Scale bar in 100 μm).

The effects of engineered extracellular vesicles on satellite cells in mice were examined through *in situ* injections. An *in situ* knockdown model of miR-140-5p in the gastrocnemius muscle was established using adeno-associated virus (AAV-KO-140) to verify the effects of miR-140-5p on satellite cells. The results showed a significant decrease in miR-140-5p content in the MPC-Exo^140−^ group after exosome injection. In the AAV-KO-140 group, where a sponge method was used to directly affect the downstream genes of miR-140-5p, the expression was not significantly different from that in the control group, nor was it different from that in the muscle-derived extracellular vesicle group (Figure 4A). After three injections, *Pax7* and its downstream gene *MyoD* significantly increased in the remaining groups compared to the control group that received physiological saline only (Figure 4B, 4C, 4E). Immunofluorescence analysis showed that under normal conditions, satellite cells remained in a resting state, located within the cell pool between muscle fibers, and only underwent self-renewal within the cell pool. However, after three *in situ* injections of MPC-Exo and MPC-Exo^140−^, the number of *Pax7^+^*/*MyoD^+^* cells significantly increased. Similarly, in the AAV-KO-140 model, a significant increase in the number of *Pax7^+^*/*MyoD^+^* cells was observed, indicating significant activation of satellite cells (Figure 4F). Notably, in the AAV-KO-140 group, the expression of *myogenin*, which is responsible for the myogenic differentiation of satellite cells, significantly decreased, suggesting that after AAV-KO-140, the high expression of *Pax7* activated satellite cells, promoting their proliferation while significantly inhibiting their differentiation (Figure 4D).

### Muscle injection of MPC-Exo^140−^ promotes skeletal muscle regeneration

**Figure 5:**
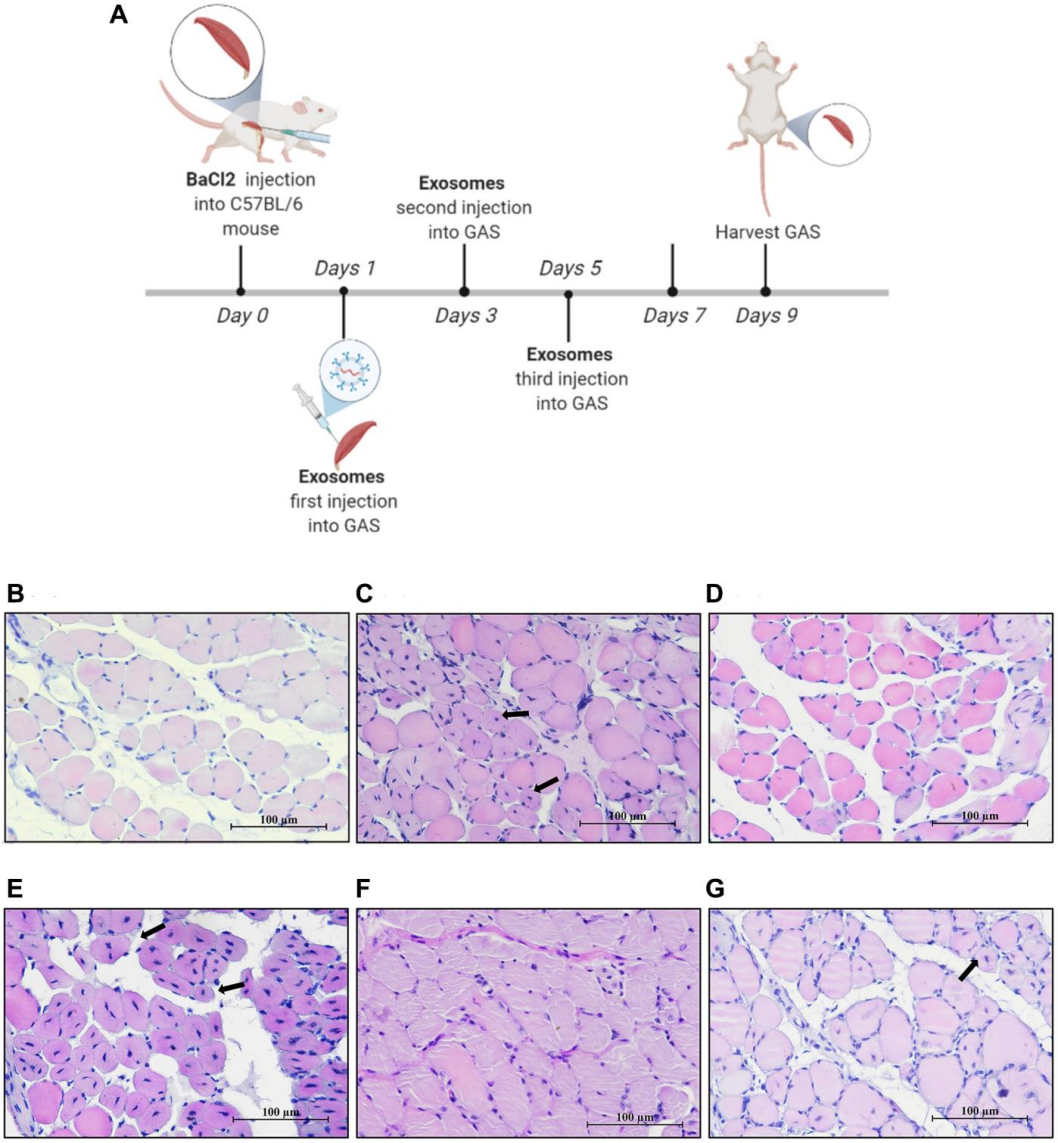
**MPC-EXO and MPC-Exo^140−^ advanced the repair of gastrocnemius muscle after acute injury.** Eight-week-old male wildtype C57BL/6 mice were treated with MPC-Exo, MPC-Exo^140+^ and MPC-Exo^140−^ 3 times after acute injury. (**A**) presents a schematic diagram of muscle injury modeling treated with MPC-EVs. (**B**–**G**) depict the HE staining results after three treatment sessions, with arrows pointing to central nuclei. The control group (**B**) shows closely arranged muscle cells with nuclei at the cell periphery. The injury group (**C**) presents newly formed muscle cells with centrally located nuclei and a few inflammatory cells between cells. In the MPC-Exo intervention group (**D**), the nuclei of newly formed muscle cells migrated toward the cell membrane. The MPC-Exo^140+^ intervention group (**E**) shows numerous centrally located nuclei in newly formed muscle cells and a loose extracellular matrix structure. In the MPC-Exo^140−^ intervention group (**F**), the muscle cells were closely arranged, and the nuclei of newly formed muscle cells moved to the cell periphery. The AAV-KO-140 injury group (**G**) shows newly formed muscle cells with nuclei relocated to the cell periphery, but the fusion process between cells and newly formed multinucleated myotubes is inhibited, resulting in a significant increase in the number of nuclei (Scale bar in 100 μm).

To further validate the therapeutic effect of MPC-Exo^140−^ on muscle-injured mice, we administered injections of 50 μL of MPC-EVs at a concentration of 2.3E+10 particles/mL into the gastrocnemius muscle of the injured mice every other day for a total of 5 days. Concurrently, to investigate the role of muscle-derived extracellular vesicles and miR-140-5p in muscle repair, we utilized an adeno-associated virus to knock down miR-140-5p in the model. The mice underwent BaCl_2_-induced muscle injury, followed by injections of the same dose of PBS every other day (Figure 5A). We collected samples from the five groups of mice every other day after the injury and utilized HE staining, immunofluorescence IF, qPCR, and WB to assess the effects of the engineered extracellular vesicles on muscle repair.

Morphological analysis revealed that by the ninth day after the injury, unlike normal mouse muscles (Figure 5B), the nuclei of newly formed muscle cells in the injured mice were primarily located in the center of the cells, indicating an ongoing repair process (Figure 5C). In the group that received MPC-Exo injections, the nuclei of the newly formed muscle cells had moved toward the cell membrane, indicating that the repair process was nearing completion (Figure 5D). In contrast, in the group that received MPC-Exo^140+^ injections, a significant number of central nuclei were still present in the newly formed muscle cells, suggesting that MPC-Exo^140+^ counteracted the repair effect of MPC-Exo (Figure 5E). However, in the group that received MPC-Exo^140−^ injections, the muscle cells exhibited close arrangement, and the nuclei of the newly formed muscle cells had moved to the cell membrane, indicating complete repair (Figure 5F). Additionally, nine days after the injury, in the group with adeno-associated virus-induced knockdown of miR-140-5p, although a few central nuclei were still observed, most of the nuclei of the newly formed muscle cells had moved to the cell membrane, indicating advanced stages of repair (Figure 5G).

**Figure 6:**
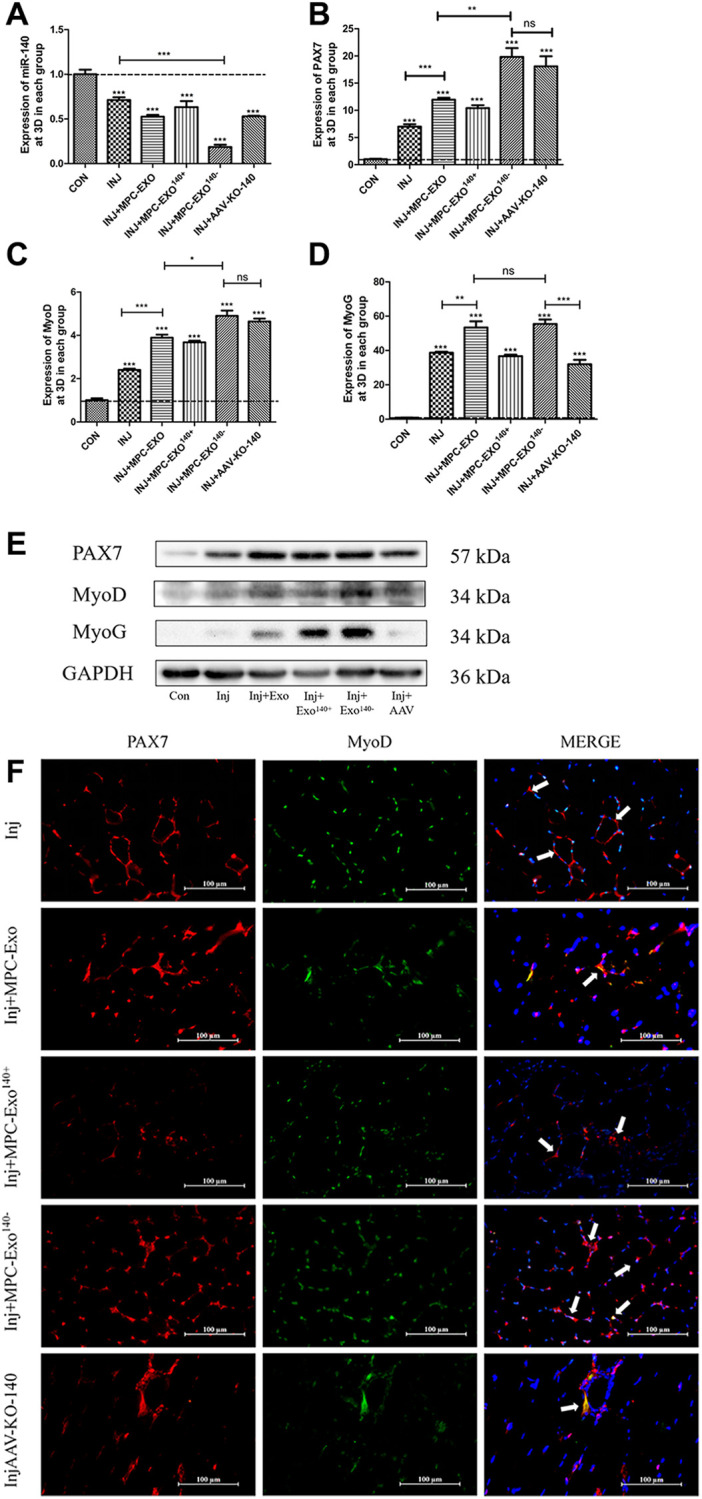
**MPC-Exo and MPC-Exo^140−^ activate muscle satellite cells to promote repair of gastrocnemius muscle injury.** (**A**–**C**) and (**D**) show the mRNA expression changes (fold change) of miR-140-5p and muscle regeneration-related factors *Pax7*, *MyoD*, and *myogenin* after two interventions with MPC-EVs on the injured muscle. (**E**) presents changes in protein expression. (**F**) displays the impact of MPC-EVs on muscle satellite cells postinjury: activated SCs are characterized by the coexpression of *Pax7^+^*/*MyoD^+^* (Figure 4F). The marked locations in the figure show that in the injury group, SCs enter an activated state stimulated by stress signals and move from the cell pool to between damaged muscle fibers. In the injected myogenic extracellular vesicle group, there was a significant increase in activated SCs around the muscle fibers. In the injected MPC-Exo^140+^ group, miR-140-5p mimics antagonize the activation effect of MPC-Exo on SCs, resulting in a significant decrease in the number of *Pax7^+^*/*MyoD^+^* cells. In the injected MPC-Exo^140−^ group and the AAV-KO-140 group, there was a significant increase in activated SCs. This suggests that under the intervention of miR-140-5p inhibitors, *Pax7* and its downstream gene *MyoD* are activated in SCs, promoting SC proliferation (Scale bar in 100 μm). Different letters between bars mean *P* ≤ 0.05 analyses followed by non-paired Student’s *t*-test. ^ns^*p* > 0.05, ^*^p < 0.05, ^**^p < 0.01 and ^***^p < 0.001 vs. CON.

To investigate the roles of the aforementioned engineered extracellular vesicles and adeno-associated virus in skeletal muscle injury repair, we examined the location and expression changes of the satellite cell marker gene *Pax7*, as well as the muscle regeneration-related proteins *MyoD* and *myogenin*. The results showed that on the third day after injury, the expression levels of *Pax7*, *MyoD*, and *myogenin* significantly increased in the group injected with MPC-Exo^140-^ compared to the injured group and the MPC-Exo group (Figure 6A–6E). In the MPC-Exo^140−^ group, the number of *Pax7^+^*/*MyoD^+^* cells significantly increased, indicating significant activation of satellite cells and promotion of muscle repair. Moreover, compared to the injection of muscle-derived extracellular vesicles, the repair process was accelerated in the MPC-Exo^140−^ group (Figure 6F).

### Intramuscular injection of MPC-Exo promotes muscle fiber regeneration and slow-to-fast fiber-type transition in the gastrocnemius muscle

**Figure 7:**
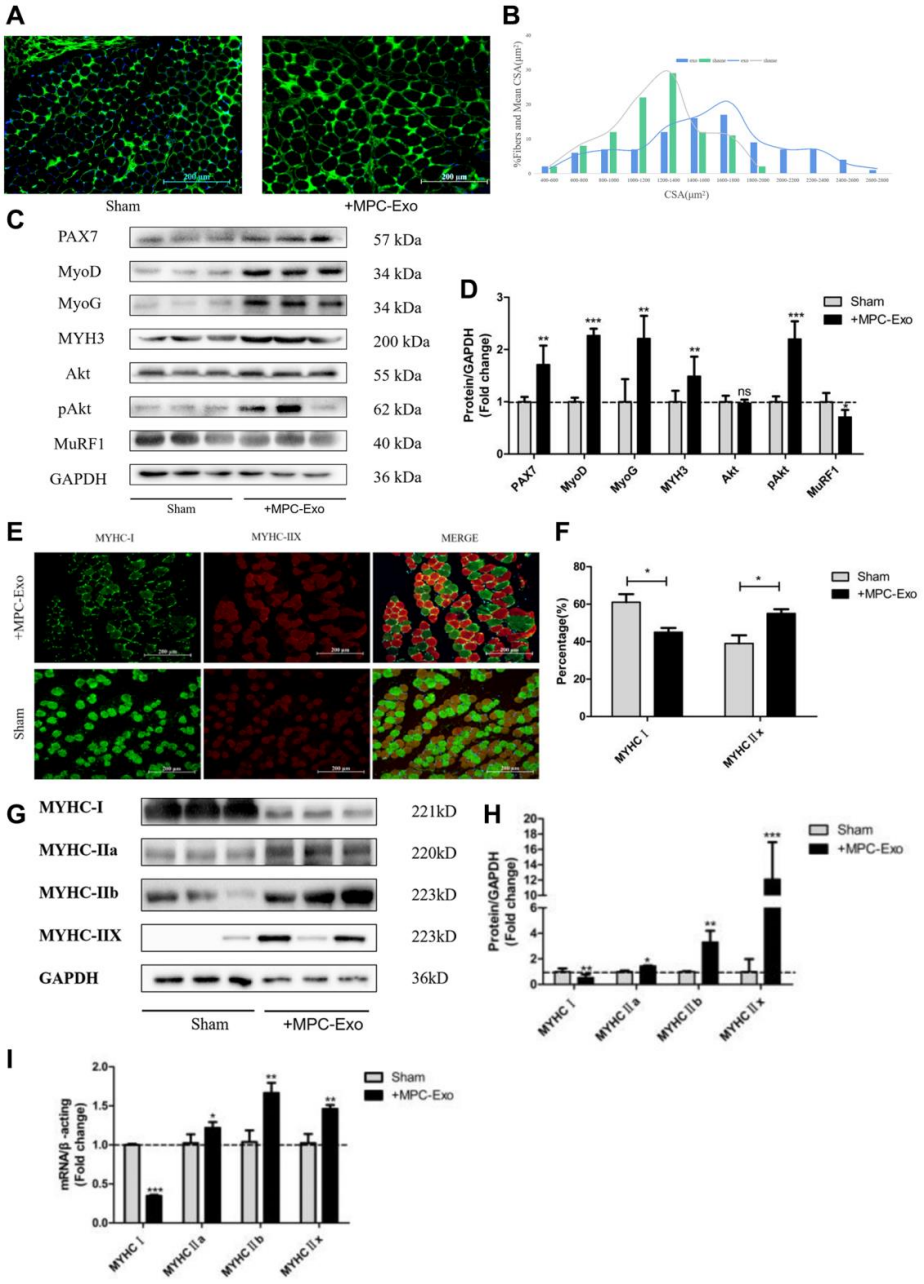
**MPC-Exo promote muscle development by activating satellite cells and fiber type transformation.** Eight-week-old male wildtype C57BL/6 mice were treated with MPC-Exo 3 times. (**A**) uses laminin immunofluorescence staining to mark the changes in the cross-sectional area of the gastrocnemius muscle after injection (Scale bar in 200 μm). (**B**) presents a statistical analysis of its fiber area frequency distribution. (**C**, **D**) show WB results, revealing increased expression of muscle regeneration-related proteins and muscle protein synthesis metabolic proteins, as well as decreased expression of muscle protein breakdown metabolic proteins after three injections. (**E**) and (**F**) show IF results, indicating a decrease in MyHC I (green fluorescence) and an increase in MyHC IIx (red fluorescence) after injection (Scale bar in 100 μm). (**G**–**I**) show WB and RT-PCR results, indicating a transition of muscle fiber type from slow-twitch to fast-twitch. Different letters between bars mean *P* ≤ 0.05 analyses followed by non-paired Student’s *t*-test. ^ns^*p* > 0.05, ^*^p < 0.05, ^**^p < 0.01 and ^***^p < 0.001 vs. Sham.

In our previous studies, we observed that MPC-Exo stimulated the proliferation and differentiation of satellite cells. To validate this finding, we measured the cross-sectional area of gastrocnemius muscle fibers in mice following MPC-Exo injections. We found that after three injections, the cross-sectional area of gastrocnemius muscle fibers significantly increased in the experimental group (Figure 7A). The overall size distribution of gastrocnemius muscle fibers in the sham-operated group and the group injected with MPC-Exos is displayed in the figure. Analysis of the data revealed that injecting myogenic extracellular vesicles resulted in a significant increase in the percentage of large-diameter fibers (shifted to the right) (Figure 7B).

To determine if the increase in muscle fiber cross-sectional area reflected an enhanced response to muscle regeneration by myogenic extracellular vesicles, we examined the expression of muscle regeneration protein markers in the gastrocnemius muscle after three injections. We observed that the expression of muscle regeneration-related proteins, including myoD, myogenin, and eMyHC, increased in the gastrocnemius muscle following the injections (Figure 7C, 7D). Additionally, the phosphorylated expression of Akt, a protein related to muscle protein synthesis, was strengthened, while the expression of TRIM63/MuRF1, a protein involved in muscle degradation metabolism, decreased.

Furthermore, the results indicated that after intervention with myogenic extracellular vesicles, the proportion of MyHC I-type muscle fibers decreased, while the proportion of MyHC IIx-type muscle fibers increased (Figure 7E–7H). This suggests a transition in the type of gastrocnemius muscle fibers from slow-twitch fibers (MyHC I) to fast-twitch fibers (MyHC IIa, MyHC IIb, MyHC IIx).

## DISCUSSION

Muscle repair commonly involves the activation and transformation of inflammatory cells, proliferation of interstitial base cells, and activation, proliferation, and differentiation of satellite cells, which play a vital role. Traditional satellite cell transplantation, which has been widely researched over past decades, involves isolating healthy satellite cells or other stem cells from donor muscles and transplanting them into the muscles of afflicted hosts. This forms hybrid fibers that correct partial muscle dystrophy or injury. However, this technology encounters many technical challenges, such as the need for immunosuppression and a significant number of satellite cells [25–29]. An effective method for enhancing muscle regeneration in diseases is to boost the endogenous myogenic potential of satellite cells [30]. Injecting Wnt7a or p38, JAK/STAT inhibitors into the muscles can increase the number of satellite cells, muscle fiber size, and muscle strength in a Duchenne muscular dystrophy mouse model [31–34].

Our understanding from current research is that, following damage to muscle structure and integrity, skeletal muscle can rapidly regenerate into mature muscle fibers. This regenerative capacity hinges on the myogenic potential of muscle satellite cells, which is regulated by various intracellular genes, among which *Pax7* plays a significant role. As a muscle satellite cell marker, *Pax7* activates quiescent satellite cells and encourages their proliferation during muscle repair [35]. We have confirmed that *Pax7* is one of the regulatory targets of miR-140-5p in satellite cell proliferation and differentiation and can be activated by suppressing miR-140-5p, thereby promoting the activation and proliferation of satellite cells.

In our team’s prior studies, we obtained myogenic progenitor cell exosome MPC-Exo and found that these vesicles stimulate skeletal muscle regeneration, speed up adipogenesis in damaged muscles, and inhibit fibrosis in damaged muscles, thus hastening injury repair [23]. By using the high biocompatibility and low immunogenicity of natural extracellular vesicles, we loaded miR-140-5p mimics/inhibitors into MPCs [36–38]. Our findings indicated that, compared to standard MPCs, MPC-Exo-140 further activated muscle satellite cells and hastened their proliferation and differentiation. This suggests that MPC-Exo-140 stimulates the activation of muscle satellite cells by activating *Pax7* and its downstream genes.

In therapeutic terms, administering MPC-Exo^140−^ accelerated the repair of the gastrocnemius muscle in mice with acute muscle injuries. Muscle regeneration can be divided into several stages, each characterized by different MRF expression. In the resting phase, satellite cells are inactive but remain in an activated state to maintain self-renewal within the cell pool, with low *Pax7* expression. When muscles are damaged, satellite cells migrate to the injury site, *Pax7* expression increases, and they reenter the cell cycle to proliferate. At this stage, related satellite cells are referred to as myoblasts. Following this, myoblasts exit the cell cycle, *Pax7* and *Myf5* expression diminishes, and the levels of *myogenin* and MRF4 increase, indicating the necessity for *Pax7* fine-tuning [30, 39, 40]. We activated the proliferative potential of satellite cells by using an adenovirus to suppress miR-140-5p, but a high concentration of *Pax7* inhibited *myogenin* expression, slowing its myogenic differentiation [41]. In this context, administering engineered extracellular vesicles during the repair stage swiftly regulated the expression of related genes without inhibiting the differentiation of satellite cells into myoblasts, accelerating injury repair. However, the underlying mechanism still requires further investigation.

**Figure 8:**
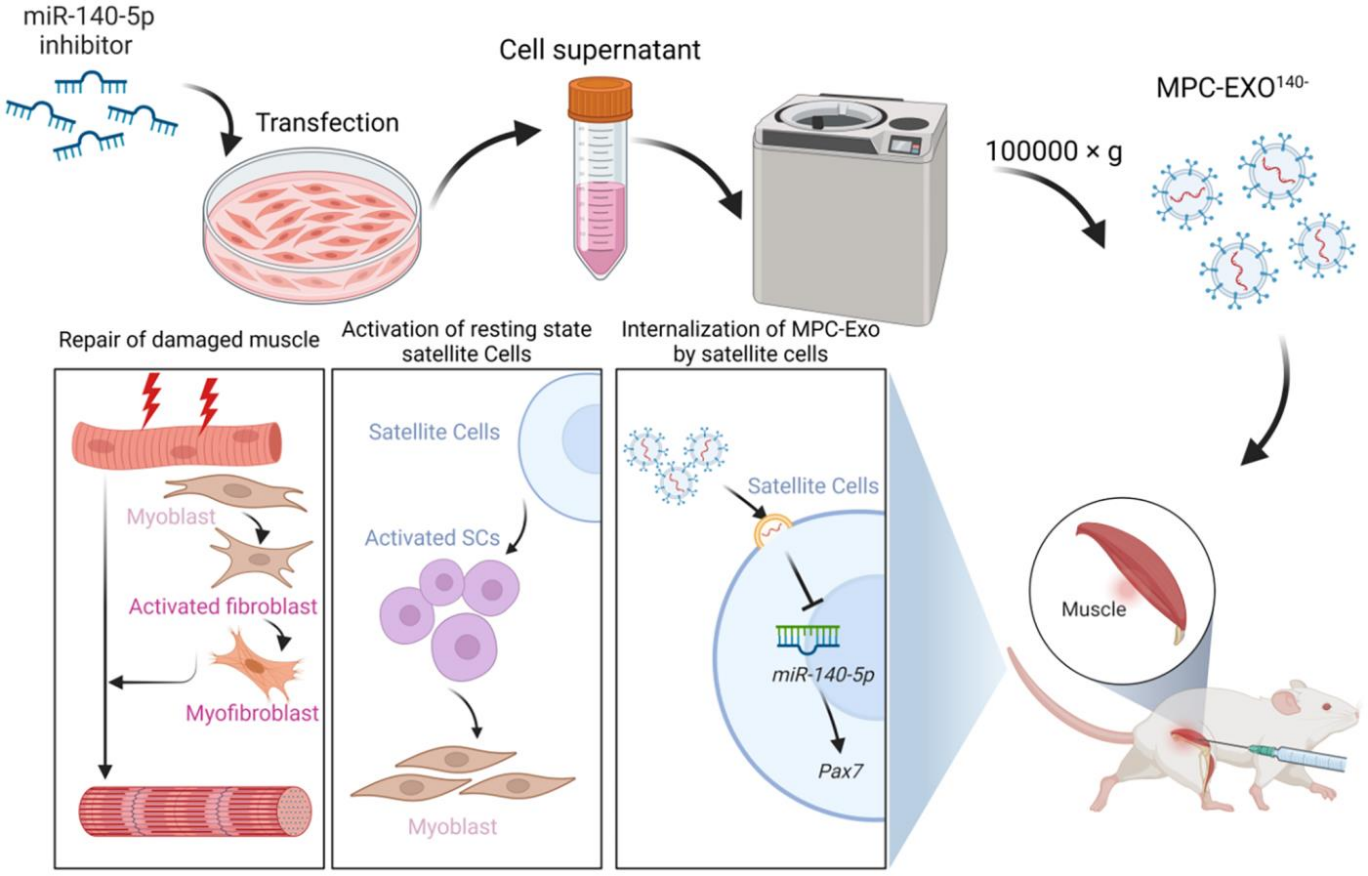
MPC-Exo^140−^ can activate dormant muscle satellite cells, initiating their proliferation and differentiation processes, ultimately leading to the formation of new skeletal muscle cells and promoting skeletal muscle repair and remodeling.

The study reveals that myogenic extracellular vesicles are crucial mediators for establishing cellular communication within muscle tissue. They can selectively carry genetic materials related to muscle development, such as miR-140-5p, and target surrounding cells, thereby influencing gene expression within SCs, affecting the activation of satellite cells, and ultimately participating in the regeneration and repair of skeletal muscles after injury (Figure 8).

## METHODS

### Animal experiments

C57BL/6J mice were obtained from Vital River (Beijing, China). All animal experiments were conducted in compliance with the approved protocol (SXAU-EAW-2021M.001227) by the Animal Care Committee of Shanxi Agricultural University. Four-week-old C57BL/6J mice were used for primary muscle satellite cell (SC) isolation, while eight-week-old mice were used for the establishment of injury and treatment models. The mice were housed under specific pathogen-free conditions with a 12:12 hour light-dark cycle.

The design, production, and validation of the adeno-associated virus knockout vector (Supplementary Material and Destination carrier and sequence information in Supplementary Figure 1) were performed by Hanheng (Wuhan, Hubei, China). Five-week-old mice were injected with adeno-associated virus carrying miR-140-5p inhibitors and control plasmids (1.8 × 10^12^ vg/mL, 50 μL). Three weeks later, the gastrocnemius (GAS), tibialis anterior muscles, and heart were collected, and RNA was extracted for target gene measurement.

On days 1, 3, and 5 after the induction of skeletal muscle injury using BaCl_2_, 50 μL of MPC-Exo was injected into the right GAS muscle of 8-week-old mice, while 50 μL of PBS was injected into the GAS muscle of the sham-operated group as a control.

### Isolation and characterization of myogenic progenitor cell-derived exosomes

The isolation procedure for MPC-Exos was based on our previous work [23]. For engineered exosomes, miR-140-5p mimics and inhibitors (sequence information in Supplementary Table 1, Universal, Xuzhou, Anhui, China) were transfected into cells at 60% confluence using Lipofectamine™ 2000 (Thermo Fisher Scientific, Waltham, MA, USA). At 6 hours post transfection, cells were cultured in growth medium for 24 hours and then differentiated with 2% horse serum. When C2C12 (BFN608006353, Bluefbio, Shanghai, China) cells reached 80% differentiation, Dulbecco’s modified Eagle’s medium (BI, Kibbutz-Beit Hamek, Israel) without serum was added, and the cells were starved for 36 hours. The cell supernatant was collected and centrifuged to isolate the exosomes. The isolation steps included centrifugation at 300 × g for 10 minutes, 2000 × g for 10 minutes, and 10000 × g for 30 minutes to remove cell debris from the supernatant. The supernatant was then centrifuged (Preparative Ultracentrifuges, Hitachi, Japan) at 100000 × g for 90 minutes to obtain the exosomes, which were washed with PBS buffer and centrifuged again at 100000 × g for 90 minutes. The morphology of myogenic progenitor cell extracellular vesicles (MPC-EVs) was identified by transmission electron microscopy (TEM, Hitachi, Japan) after fixation, gradient dehydration, and gold plating. The exosomal proteins TSG101 (ab125011, Abcam), CD9 (98327, CST), and CD63 (52090, CST) were detected by Western blotting. The concentration and size of the exosomes were measured using nanoparticle tracking (NanoSight NS300, Malvern Instruments, Westborough, MA, USA) analysis.

### Establishment and treatment of BaCl2-induced GAS injury

The procedure for establishing BaCl_2_-induced GAS injury was based on previous work [24]. Briefly, we used a micro sample syringe (F519162, Shenggong, China) to inject BaCl_2_ solution from one end of the gastrocnemius muscle and inject it at multiple points while advancing, 80–100 μg (0.5% of body weight) of 1.2% BaCl_2_ was injected into the right GAS muscle of the control and adeno-associated virus knockout mice on day 0. On days 1, 3, and 5 after the injury, 50 μL of MPC-EVs were injected into the right GAS muscle of the injured group, while 50 μL of PBS was injected into the GAS muscle of the sham-operated group as a control. On days 1, 3, 5, 7, and 9, one-third of the GAS muscles were collected and fixed with Bouin’s fixative for histological analysis, while the remaining samples were used for RNA and protein extraction. For hematoxylin and eosin (HE) and immunofluorescence (IF) staining, 6 μm sections were stained, and at least three fields were captured for analysis using inverted fluorescence microscopy (Nikon Eclipse Ts-2R, Tokyo, Japan).

### Cultivation of primary muscle satellite cells

The isolation procedure for primary SCs was based on our previous work [24]. Hindlimb muscles from 4-week-old mice were dissected and minced, followed by digestion with collagenase 2 (17101015, Gibco, Waltham, MA, USA) at 37°C for 30 minutes. Subsequently, digestion with collagenase D (11088858001, Roche, Basel, Switzerland) and dispase II (04942078001, Roche, Basel, Switzerland) was performed for 1 hour. The resulting cell suspension was filtered through a 45 μm cell filter. After labeling with CD31^−^/CD45^−^/Sca-1^+^/Integrin-α7^+^ antibodies (553372/55300/553336/FAB3518A, BD, San Diego, CA, USA), primary muscle satellite cells were sorted using flow cytometry (FACS Aria III, BD, San Diego, CA, USA). The sorted cells were cultured in Ham's F-10 Nutrient Mixture medium supplemented with 20% fetal bovine serum, 100 μg/mL penicillin, and 100 μg/mL streptomycin (growth medium). The isolated muscle satellite cells were passaged a maximum of 5 times.

### Flow cytometric analysis of cell cycle and cell viability assay

Cell cycle analysis was performed following the method described by Herman-Antosiewicz et al. [25]. Briefly, cells were co-incubated at 37°C for 24 hours in separate culture media and media containing different MPC-EVs. After incubation, the cells were washed with cold PBS, fixed in 70% ethanol, and stored at 4°C for subsequent cell cycle analysis. Fixed cells were then washed once with PBS and resuspended in 1 mL of propidium iodide (PI) staining solution (50 mg/mL PI and 1 mg/mL RNAse in 1 mL of sodium citrate buffer, pH 7.4). Following a 30-minute incubation in the dark, the distribution of cells in the cell cycle was measured using the cyan flow cytometry analysis system (FACS Aria III, BD, San Diego, CA, USA), and the cell cycle distribution was quantified using MultiCycle software, calculating the percentages of cells in G1, S, and G2 phases.

To evaluate the proliferation capacity of SCs, a CCK-8 assay (ab228554, Abcam, USA) was performed according to the manufacturer's instructions.

### Dual-luciferase reporter gene assay

The binding sites of miR-140-5p on *Pax7* were predicted using TargetScan (http://www.TargetScan.org/) and miRWalk (http://mirwalk.umm.uni-heidelberg.de/). The design, production, and validation of psi-CHECK2-*PAX7*-3′UTR and its mutant vectors (Supplementary Material and dual luciferase vector information in Supplementary Figure 2) were performed by Universal (Xuzhou, Anhui, China). The plasmids and miR-140-5p mimics were cotransfected into HEK293T cells using Lipofectamine™ 2000. After 48 hours, the cells were harvested, and luciferase activity was measured using a dual-luciferase reporter assay kit (11402ES60, Yeasen, China) following the manufacturer's protocol.

### *In vitro* phagocytosis experiment

Exosomes were labeled with PKH67 dye (PKH67GL, Sigma, GER, USA) following the manufacturer’s instructions. Briefly, 5 × 10^−6^ M PKH67GL dye was added to 1 μg of EVs and incubated for 5 minutes. The staining reaction was stopped by adding 2 mL of FBS for 1 minute. The labeled exosomes were then washed with complete culture medium and centrifuged at 100000 g and 4°C for 30 minutes. After washing with complete medium, the exosomes were centrifuged again at 100000 g and 4°C for 1 hour. The particles were resuspended in 100 μL of PBS. The solution containing labeled exosomes was transferred to a 10 kDa MWCO (UFC9010, Millipore, USA) retention column and centrifuged at 4°C and 3000 × g for 40 minutes. The concentrate was collected and stored in a dark box at 4°C.

For the *in vitro* phagocytosis experiment, PKH67-labeled MPC-EVs were added to SCs and incubated for 3 hours. Subsequently, the cells were fixed with 4% paraformaldehyde for 30 minutes and coverslipped with an anti-fade reagent containing DAPI (S2110, Solarbio, China). Fluorescence images were captured using a laser scanning confocal microscope (Olympus, Tokyo, Japan).

### Protein blotting and antibodies

Protein blotting was performed using the equal mass method, with 50 μg of protein used in cell experiments and 20 μg *in vivo* experiments. The primary antibodies used include MYH3 (22287-1-AP, Proteintech, China), TSG101 (ab30871, Abcam, USA), CD9 (ab223052, Abcam), CD63 (ab213090, Abcam), *Pax7* (ab199010, Abcam), MyoD (TA7733M, Abmart, China), MyoG (sc-12732, Santa, USA), Akt (4691S, CST, USA), p-Akt (4060S, CST), MuRF1 (55456-1-AP, Proteintech, China), MYH7 (22280-1-AP, Proteintech), MYH4 (20140-1-AP, Proteintech), MYH2 (ab124937, Abcam), MYH1 (67299-1-Ig, Proteintech), GAPDH (HRP-60004, Proteintech). Protein bands were scanned and quantified using the ChemiDoc XRS+ gel imaging system (Bio-Rad, Hercules, CA, USA).

### RNA extraction and real-time qPCR

Total RNA from blood was isolated using RNAiso blood (9112, Takara, Japan), and total RNA from exosomes was isolated using the miRNeasy Serum/Plasma Kit (217184, Qiagen, USA). The concentration of RNA was measured using a NanoDrop One spectrophotometer (Thermo Fisher Scientific, USA). cDNA synthesis was performed using the PrimeScript™ RT Reagent Kit with gDNA Eraser (RR047A, Takara, Japan), and the miRNA 1st Strand cDNA Synthesis Kit (MR101, Vazyme, China) by stem-loop was used for miRNA cDNA synthesis. Real-time qPCR was performed using ChamQ SYBR qPCR Master Mix (Q331, Vazyme). The primer sequences used are shown in Supplementary Table 1.

### Immunofluorescence and microscopy

Six-micrometer slices of the gastrocnemius muscle were subjected to antigen retrieval with sodium citrate buffer (C1032, Solarbio, China). Background blocking was performed with 10% goat serum (SL038, Solarbio) at room temperature for 10 minutes, followed by overnight incubation with primary antibodies (Laminin, PA1-16730, Thermo Fisher Scientific, USA; *Pax7*, 20570-1-AP, Proteintech, China; *MyoD*, TA7733M, Abmart, China; MYH7, 22280-1-AP, Proteintech, China; MYH1, 67299-1-Ig, Proteintech, China) at 4°C. The samples were then washed with PBS and incubated with Alexa Fluor 488 and 594 fluorescent secondary antibodies (ab150077, ab150116, Abcam) at room temperature for 1 hour. After washing, the samples were coverslipped with an anti-fade reagent containing DAPI. Fluorescence images were captured using an inverted fluorescence microscope (Nikon Eclipse Ts-2R, Japan).

### Statistical analysis

All statistical data are presented as the mean ± standard deviation (SD). Comparisons between two groups were performed using two-tailed Student’s *t*-test, and comparisons among multiple groups were performed using one-way analysis of variance (ANOVA) with Tukey’s post hoc test. A *p*-value of less than 0.05 (*P* < 0.05) is considered statistically significant. All statistical analyses were conducted using SPSS 23.0, and the graphs were created using GraphPad Prism 5.0.2.

## Supplementary Materials

Supplementary Material

Supplementary Figures

Supplementary Table 1
