# Supplementary material for: Myogenic exosome miR-140-5p modulates skeletal muscle regeneration and injury repair by regulating muscle satellite cells
Source: Aging (Albany NY). 2024 Feb 29;16(5):4609–30. doi: 10.18632/aging.205617 (PMC10968704; doi:10.18632/aging.205617)
Supplement: Supplementary Material [file aging-16-205617-s001.pdf]

SUPPLEMENTARY MATERIAL

AAV vector and target gene information

Destination carrier information

See Supplementary Figure 1.

Target gene sequence information

mmu-miR-140-5p MIMAT0000151: CAGUGGUUUUACCCUAUGGUAG

Change U to T: CAGTGGTTTTACCCTATGGTAG

Reverse complementary mutation sequence: CTACCATAGGTACAACCACTG

Sponge structure (reverse complementary sequence concatenation after four mutations):
CTACCATAGGTACAACCACTGtatacCTACCATAGGTACAACCACTGacatcCTACCATAGGTACAACCACTG
tcttcaCTACCATAGGTACAACCACTG

Construction of dual luciferase vector

Vector plasmid map

See Supplementary Figure 2.

Sequence information

Gene name: Pax7-Mut **Sequence length:** 435

Pax7-Mut Sequence:

caca
4081 gctgcaagca cccctttcaa agaccaaatg catcctcctc cacattcctt gctccctgga
4141 ggcctggctc tggatacacc tgagtcttcg ttcacctact acactttagg agcaggaact
4201 tcaagcaggt gacatccaca gggcccagtc ccagccaagg gagcaacatt ccaacgcttg
4261 gaccaatcat aatgatgacg ggcactccca ttggcgtact agagacctgc ttgggagaaa
4321 acaaaatgac ttctcattcc atgccatgcc tctgaatgct cccccaagct gccatcttgg
4381 tataaaatgg gacttgtgtt gtggggaacc ccttgacccc aacaggtttt cccaactgtc
4441 tcatgctttt gtgaatctgt ctgctttgat ctgtaaaact cagccttgtt tgggcag

Gene name: Pax7-WT **Sequence length:** 435

Pax7-WT Sequence:

caca

4081 gctgcaagca cccctttcaa agaccaaatg catcctcctc cacattcctt gctccctgga
4141 ggcctggctc tggatacacc tgagtcttcg ttcacctact acactttagg agcaggaact
4201 tcaagcaggt gacatccaca gggcccagtc ccagccaagg gagcaacatt ccaacgcttg
4261 gaccaatcat aatgatctgc ccgtgagggt aaccgcaact agagacctgc ttgggagaaa
4321 acaaaatgac ttctcattcc atgccatgcc tctgaatgct cccccaagct gccatcttgg
4381 tataaaatgg gacttgtgtt gtggggaacc ccttgacccc aacaggtttt cccaactgtc
4441 tcatgctttt gtgaatctgt ctgctttgat ctgtaaaact cagccttgtt tgggcag

Gene name: Pax7-WT **Sequence length:** 435
Pax7-WT Sequence:
caca
4081 gctgcaagca cccctttcaa agaccaaatg catcctcctc cacattcctt gctccctgga
4141 ggcctggctc tggatacacc tgagtcttcg ttcacctact acactttagg agcaggaact
4201 tcaagcaggt gacatccaca gggcccagtc ccagccaagg gagcaacatt ccaacgcttg
4261 gaccaatcat aatgatctgc ccgtgagggt aaccgcaact agagacctgc ttgggagaaa
4321 acaaaatgac ttctcattcc atgccatgcc tctgaatgct cccccaagct gccatcttgg
4381 tataaaatgg gacttgtgtt gtggggaacc ccttgacccc aacaggtttt cccaactgtc
4441 tcatgctttt gtgaatctgt ctgctttgat ctgtaaaact cagccttgtt tgggcag
