# Supplementary material for: Myogenic exosome miR-140-5p modulates skeletal muscle regeneration and injury repair by regulating muscle satellite cells
Source: Aging (Albany NY). 2024 Feb 29;16(5):4609–30. doi: 10.18632/aging.205617 (PMC10968704; doi:10.18632/aging.205617)
Supplement: Supplementary Figures [file aging-16-205617-s002.pdf]

Supplementary Figures

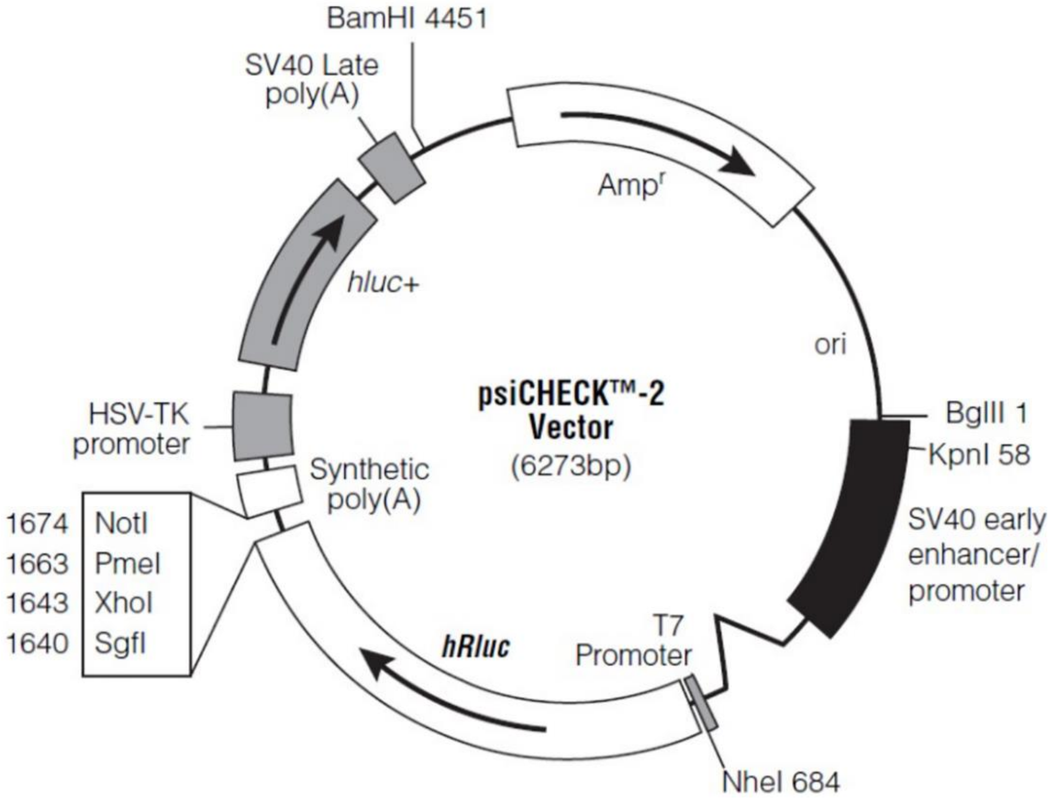

Supplementary Figure 1. pHBAAV-CMV-MCS-P2A-Luc collection of illustrative plates. We used adeno-associated virus to establish miR-140-5p knockout model, inserted the mutant sequence into pHBAAV-CMV-MCS-P2A-Luc vector, and then used adeno-associated virus to package.

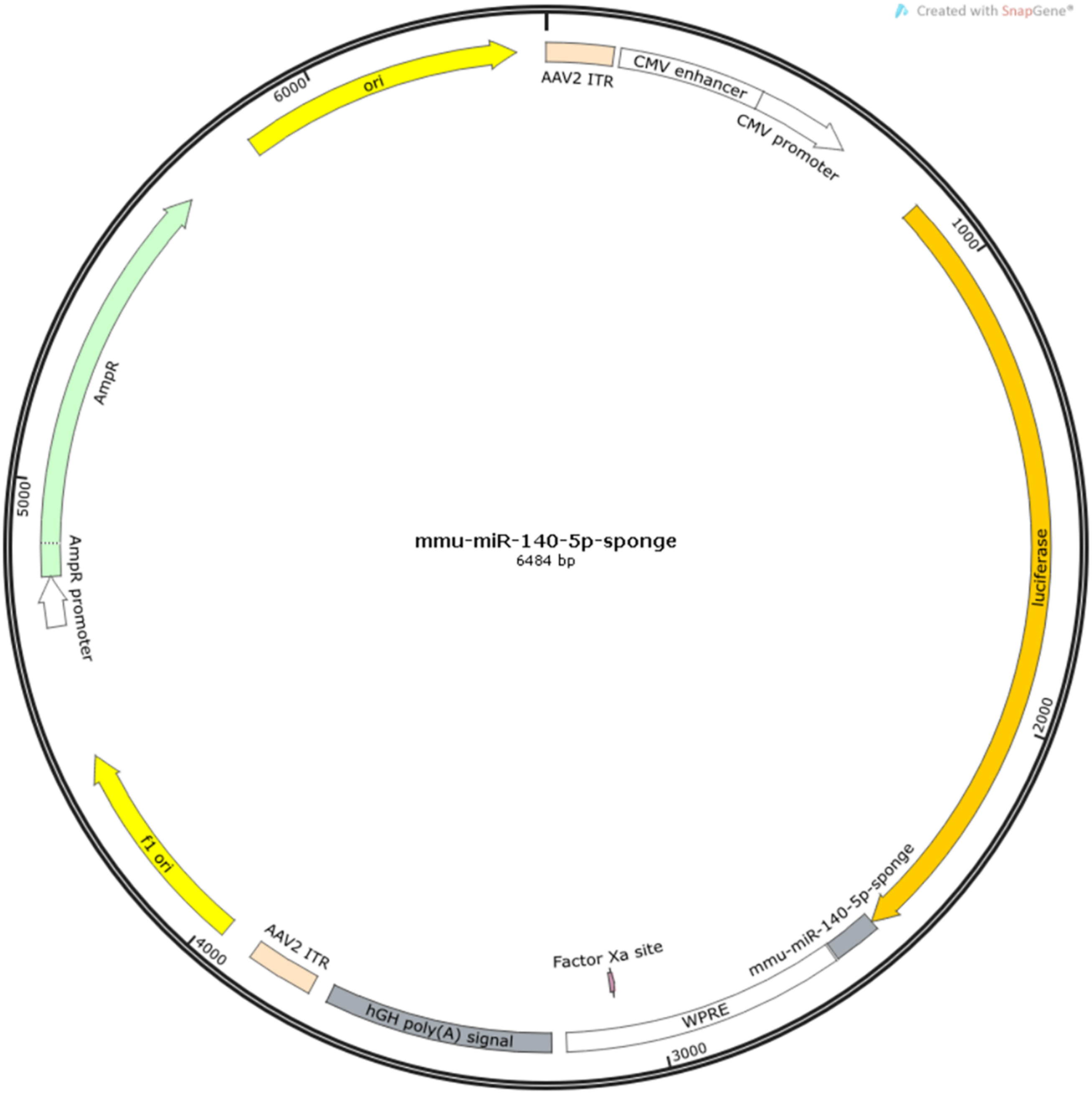

Supplementary Figure 2. PsiCHECK2 vector plasmid map. Obtained the mouse *Pax7* 3'UTR sequence from NCBI (http://www.ncbi.nlm.nih.gov/), and added 200 bases each to the binding site of miR-140-5p. They were inserted between the XhoI-NotI cleavage sites of the psiCHECK2 vector to construct a wild-type vector of *Pax7* 3'UTR; The Mutant vector of *Pax7* 3'UTR was constructed by reverse mutation of the binding site.
