# Supplementary material for: Myogenic exosome miR-140-5p modulates skeletal muscle regeneration and injury repair by regulating muscle satellite cells
Source: Aging (Albany NY). 2024 Feb 29;16(5):4609–30. doi: 10.18632/aging.205617 (PMC10968704; doi:10.18632/aging.205617)
Supplement: Supplementary Table 1 [file aging-16-205617-s003.pdf]

Supplementary Table

Supplementary Table 1. Sequences of the primers used in this study.

Gene primer	Primer sequence (5′–3′)
Myh1_F	ATGTTCCTGTGGATGGTCAC
Myh1_R	CTCGTTGGTGAAGTTGATGC
Myh2_F	TCAGGCTTCAGGATTTGGTG
Myh2 _R	GGATCTTGCGGAACTTGGATAG
Myh4_F	CTTGTCTGACTCAAGCCTGCC
Myh4_R	TCGCTCCTTTTCAGACTTCCG
Myh7_F	CCATCTCTGACAACGCCTATC
Myh7_R	GGATGACCCTCTTAGTGTTGAC
miR-140-5p_F	CGCGCAGTGGTTTTACCCTA
miR-140-5p_R	AGTGCAGGGTCCGAGGTATT
Pax7_F	CGTGTTTCCCATGGTTGTGTC
Pax7_R	AATCGAACTCACTGAGGGCAC
miR-140-5p-mimics_F	CAGUGGUUUUACCCUAUGGUAG
miR-140-5p-mimics_R	ACCAUAGGGUAAAACCACUGUU
miR-140-5p-inhibitor	CUACCAUAGGGUAAAACCACUG
MyoD_F	GAATGGCTACGACACCGCCTACTAC
MyoD_R	ACGGGGTCTGGGTTCCCTGTT
MyoG_F	AACTACCTTCCTGTCCACCTTC
MyoG_R	CACAGACTTCCTCTTACACACCT
miR-140-5p-loop	GTCGTATCCAGTGCAGGGTCCGAGGTATTCGCACTGGATACGACCTACCA
β-actin_F	TTGCTGACAGGATGCAGAAG
β-actin_R	ACATCTGCTGGAAGGTGGAC
